# A logistic regression model to predict long-term survival for borderline resectable pancreatic cancer patients with upfront surgery

**DOI:** 10.1186/s40644-025-00830-y

**Published:** 2025-02-05

**Authors:** Jin-Can Huang, Shao-Cheng Lyu, Bing Pan, Han-Xuan Wang, You-Wei Ma, Tao Jiang, Qiang He, Ren Lang

**Affiliations:** Hepatobiliary, Pancreas & Spleen Surgery Department, Beijing Chao-Yang Hospital, Capital Medical University, 8 Gongren Tiyuchang Nanlu, Chaoyang District, Beijing, 100020 P. R. China

**Keywords:** Borderline resectable pancreatic cancer, Risk factor, Upfront surgery, Nomogram, Logistic regression model, Machine learning

## Abstract

**Background:**

The machine learning model, which has been widely applied in prognosis assessment, can comprehensively evaluate patient status for accurate prognosis classification. There still has been a debate about which predictive strategy is better in patients with borderline resectable pancreatic cancer (BRPC). In the present study, we establish a logistic regression model, aiming to predict long-term survival and identify related prognostic factors in patients with BRPC who underwent upfront surgery.

**Methods:**

Medical records of patients with BRPC who underwent upfront surgery with portal vein resection and reconstruction from Jan. 2011 to Dec. 2020 were reviewed. Based on postoperative overall survival (OS), patients were divided into the short-term group (≤ 2 years) and the long-term group (> 2 years). Univariate and multivariate analyses were performed to compare perioperative variables and long-term prognoses between groups to identify related independent prognostic factors. All patients are randomly divided into the training set and the validation set at a 7:3 ratio. The logistic regression model was established and evaluated for accuracy through the above variables in the training set and the validation set, respectively, and was visualized by Nomograms. Meanwhile, the model was further verified and compared for accuracy, the area under the curve (AUC) of the receiver operating characteristic curves (ROC), and calibration analysis. Then, we plotted and sorted perioperative variables by SHAP value to identify the most important variables. The first 4 most important variables were compared with the above independent prognostic factors. Finally, other models including support vector machines (SVM), random forest, decision tree, and XGBoost were also constructed using the above 4 variables. 10-fold stratified cross-validation and the AUC of ROC were performed to compare accuracy between models.

**Results:**

104 patients were enrolled in the study, and the median OS was 15.5 months, the 0.5-, 1-, and 2- years OS were 81.7%, 57.7%, and 30.8%, respectively. In the long-term group (*n* = 32) and short-term group (*n* = 72), the overall median survival time and the 1-, 2-, 3- years overall survival were 38 months, 100%, 100%, 61.3% and 10 months, 38.9%, 0%, 0%, respectively. 4 variables, including age, vascular invasion length, vascular morphological malformation, and local lymphadenopathy were confirmed as independent risk factors between the two groups following univariate and multivariate analysis. The AUC between the training set (*n* = 72) and the validation set (*n* = 32) were 0.881 and 0.875. SHAP value showed that the above variables were the first 4 most important. The AUC following 10-fold stratified cross-validation in the logistic regression (0.864) is better than SVM (0.693), random forest (0.789), decision tree (0.790), and XGBoost (0.726).

**Conclusion:**

Age, vascular invasion length, vascular morphological malformation, and local lymphadenopathy were independent risk factors for long-term survival of BRPC patients with upfront surgery. The logistic regression model plays a predictive role in long-term survival and may further assist surgeons in deciding the treatment option for BRPC patients.

## Introduction

Pancreatic ductal adenocarcinoma (PDAC) is one of the most intractable and lethal malignancies, and is the 7th leading cause of global cancer deaths worldwide [1]. Despite significant advances in the diagnosis and treatment of PDAC, the incidence and mortality remain increasing and are projected to have a dismal prognosis and low resection rate [2]. The resectability of PDAC is primarily determined by the degree of invasion of the major artery and the portal vein [3]. The National Comprehensive Cancer Network (NCCN) guideline classifies PDAC resectability into three categories based on the degree of tumor vascular contact resectable, borderline resectable, and locally advanced disease. Among them, borderline resectable pancreatic cancer (BRPC) is fundamentally different from resectable pancreatic cancer in that it has a higher risk of positive resection margins, worse survival outcomes, more complex surgical resection procedure, and is associated with the presence of occult distant metastasis. The NCCN guidelines recommend neoadjuvant chemotherapy (NAC) for BRPC purporting to increase the probability of margin-negative resection and facilitate early treatment of hidden micro-metastatic disease without delay from waiting to recover after surgery, although there is currently insufficient high-quality evidence to conclusively demonstrate that NAC achieves superior outcomes in BRPC patients [4–6]. No objective responses or significant toxicities might further affect the prognosis and quality of life of cancer patients [7, 8].

Multiple factors may be applied to predict the long-term prognosis for cancer patients. An increasing number of studies have shown that systemic immune inflammation amongst cancer patients is closely related to metastasis and poor prognosis [9–11]. In clinical practice, carbohydrate antigen 19 − 9 (CA19-9) is the most common tumor marker for PDAC, but its value in prognosis prediction is still limited due to low sensitivity and specificity [12]. Pathological indexes, for instance, tumor size, location, lymph node metastasis, modified Glasgow prognostic score or TNM stage, and pathological grade are also applied to predict the recurrence and oversurvival of PDAC patients [13]. Serum indexes like serum alkaline phosphatase, serum bilirubin, and albumin (ALB) are also reported to be correlated with the OS of PDAC patients [14]. However, the relationship between factors and the prognosis of patients with PDAC remains controversial and needs to be further investigated. Therefore, it would be of great clinical significance to construct a reasonable and effective model to predict the survival of patients with BRPC.

Artificial intelligence (AI) and machine learning allow computers to run complex programs, which include elements of mathematics, statistics, and computer science. The application of AI and machine learning mainly benefits disease diagnosis, risk identification, and outcome prediction in the medical field. Researchers have used various AI-based machine learning models to examine diseases such as skin, liver, heart, Alzheimer’s, etc. that need to be diagnosed early [15]. So, machine learning has the potential to transform the way that medicine works.

In this paper, we reviewed the medical data of BRPC patients with upfront surgery and established logistic regression to predict long-term survival. The aim is to evaluate the predictive performance of this model and to identify risk factors related to the long-term OS for BRPC patients with upfront surgery.

## Materials and methods

### Patient selection

**Fig. 1:**
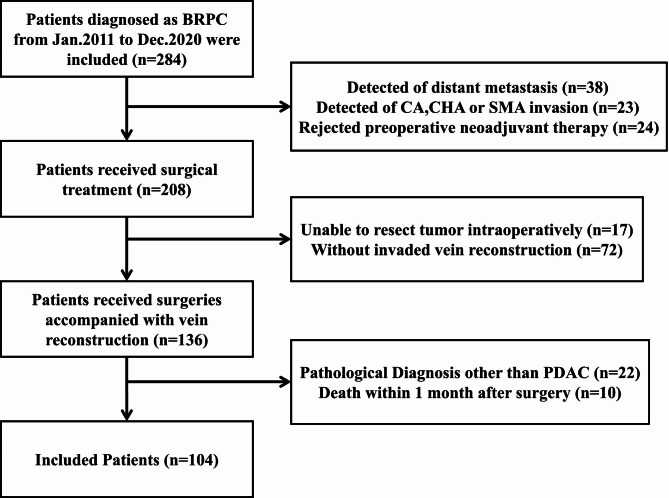
The patient selection process for the present study

Medical records of patients with BRPC who underwent upfront surgery in our hospital from Jan. 2011 to Dec. 2020 were retrospectively reviewed. The criteria for BRPC were defined by the preoperative resectable status of the National Comprehensive Cancer Network (NCCN) guidelines Version 2.2021 [3]. The invaded length, angle, and morphological malformation of the portal vein system were evaluated by preoperative contrast-enhanced imaging. The procedures for pancreatic surgery consisted of pancreaticoduodenectomy (PD), or total pancreatectomy (TP), and standard lymphadenectomy, with PV resection and reconstruction. The type of PV resection and reconstruction (direct closure or end-to-end venous anastomosis with an allogeneic vein) depended on the site and extent of tumor invasion of the vein. All patients had a confirmed pathological diagnosis as PDAC. Patients with artery invasion, distant organ metastases, and NAC were excluded. The specific including and excluding criteria are summarized in Fig. 1. Informed consent was obtained from patients and their families for the surgical procedure. This study was conducted in accordance with the Declaration of Helsinki (as revised in 2013) and was approved by the Ethics Committee of Beijing Chao-Yang Hospital (No. 2020-D-302), and individual consent for this retrospective analysis was waived.

### Assessment of portal vein system invasion

**Fig. 2:**
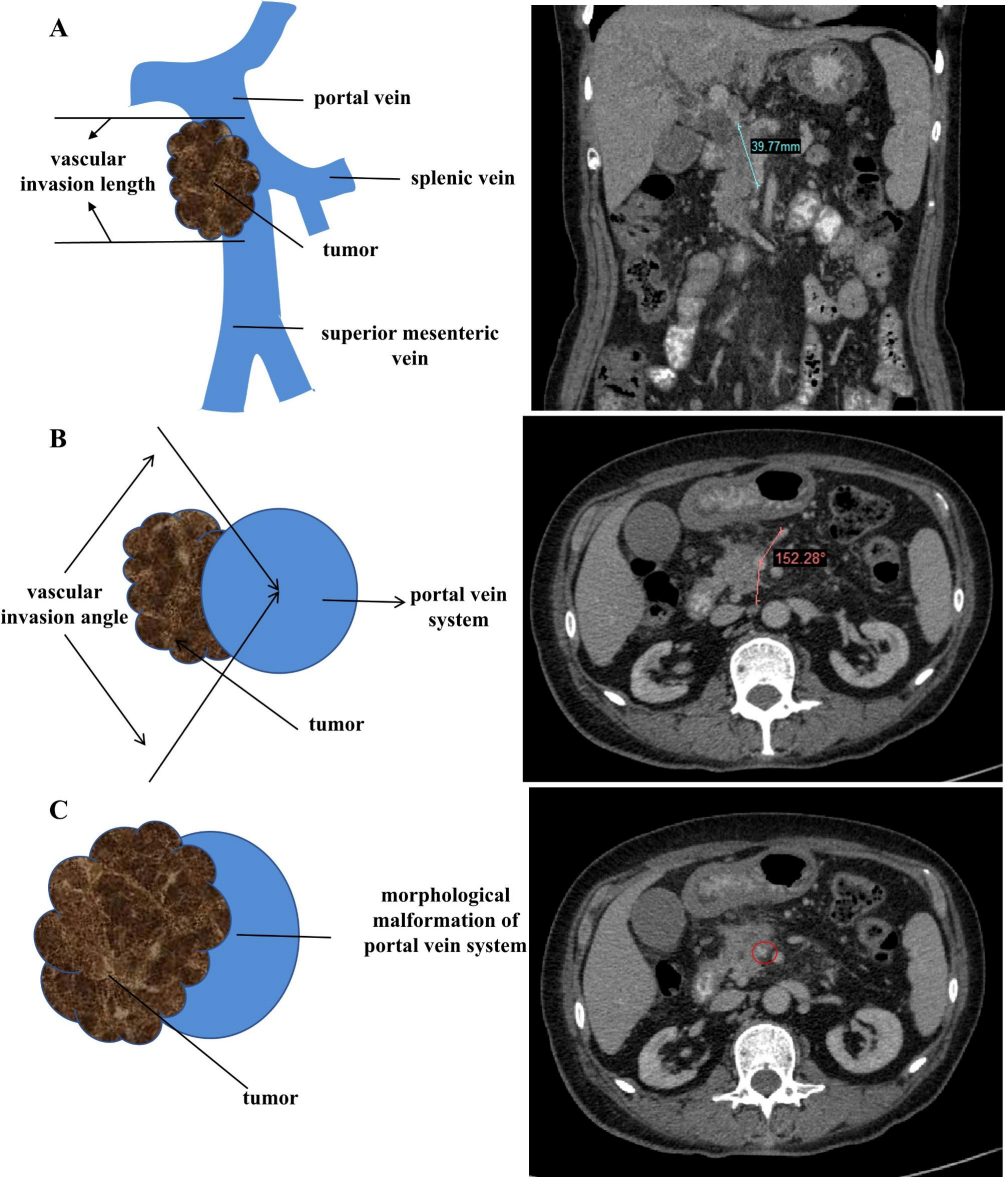
The model diagram and CT image were accordingly used to assess the portal vein system Invasion. (**A**) The length of vascular invasion measured in the coronal section. (**B**) The angle of vascular invasion was measured in the transverse section. (**C**) Vascular morphological malformation measured in transverse section

The evaluation of the invaded portal vein system was based on preoperative contrast-enhanced imaging and postoperative pathological data. Imaging assessment included the site of vein invasion, the length of vein invasion measured in the coronal section (Fig. 2A), the angle of vascular invasion measured in the transverse section (Fig. 2B), and vascular morphological malformation for instance whether the lumen of the portal vein system was narrowed and deformed (Fig. 2C).

### Patient grouping and flow chart

Based on the overall survival, patients were divided into two groups: the long-term group (≥ 2 years) and the short-term group (< 2 years). Perioperative clinical data and pathological results were analyzed and compared using univariate and multivariate analysis and SHAP values between the two groups to identify risk factors.

### Construction and validation of machine learning models

All patients in the present study were randomly divided into the training set and the validation set at a 7:3 ratio. Machine learning models were established and evaluated through the identified variables in the training set and the validation set, respectively. The Shapley additive explanations (SHAP) method was used to screen the main influencing factors of clinical data from patients. Single classification models (including decision tree, logistic regression, and support vector machine) and integrated learning algorithms (including random forest and XGBoost) were performed to predict long-term survival after surgery. The best parameters of the model were identified through 10-fold cross-validation and grid search, and the best prediction model was selected by average AUC values.

### Follow-up of patients

Patients were re-examined at the 1st month and 3rd month, every 3 months in the first 2 years after operation, and then the interval of re-examination could be changed to once a year if the results were stable. Following-up included blood examination (blood routine, blood biochemistry, tumor markers, etc.), contrast-enhanced imaging, follow-up treatment, tumor recurrence, and survival. The follow-up cut-off date was December 2022, and the endpoints of follow-up were tumor recurrence and patient death. OS was defined as the time between the date of surgery and the date of death due to any reason or the last follow-up.

### Statistical analysis

Continuous variables were summarized using the mean and standard deviation or median and interquartile range, while categorical variables were summarized using frequency and percentage. Differences between groups were analyzed using either a t-test for continuous variables or a Chi-square test for categorical variables. Survival was estimated using the Kaplan-Meier method with 95% confidence intervals, and compared using the log-rank test. All statistical tests were two-sided and differences were considered significant when *P* < 0.05. Statistical analysis was performed using SPSS (IBM 19.0) and R 4.2.2 software. We have obtained a copyright license about it. The accuracy and discriminatory power of the nomogram were evaluated using an AUC value. The calibration curve was used to assess the predictive performance of the model. The closer the curve is to the diagonal line, the stronger the predictive performance of the model.

## Results

### Patient characteristics

**Fig. 3:**
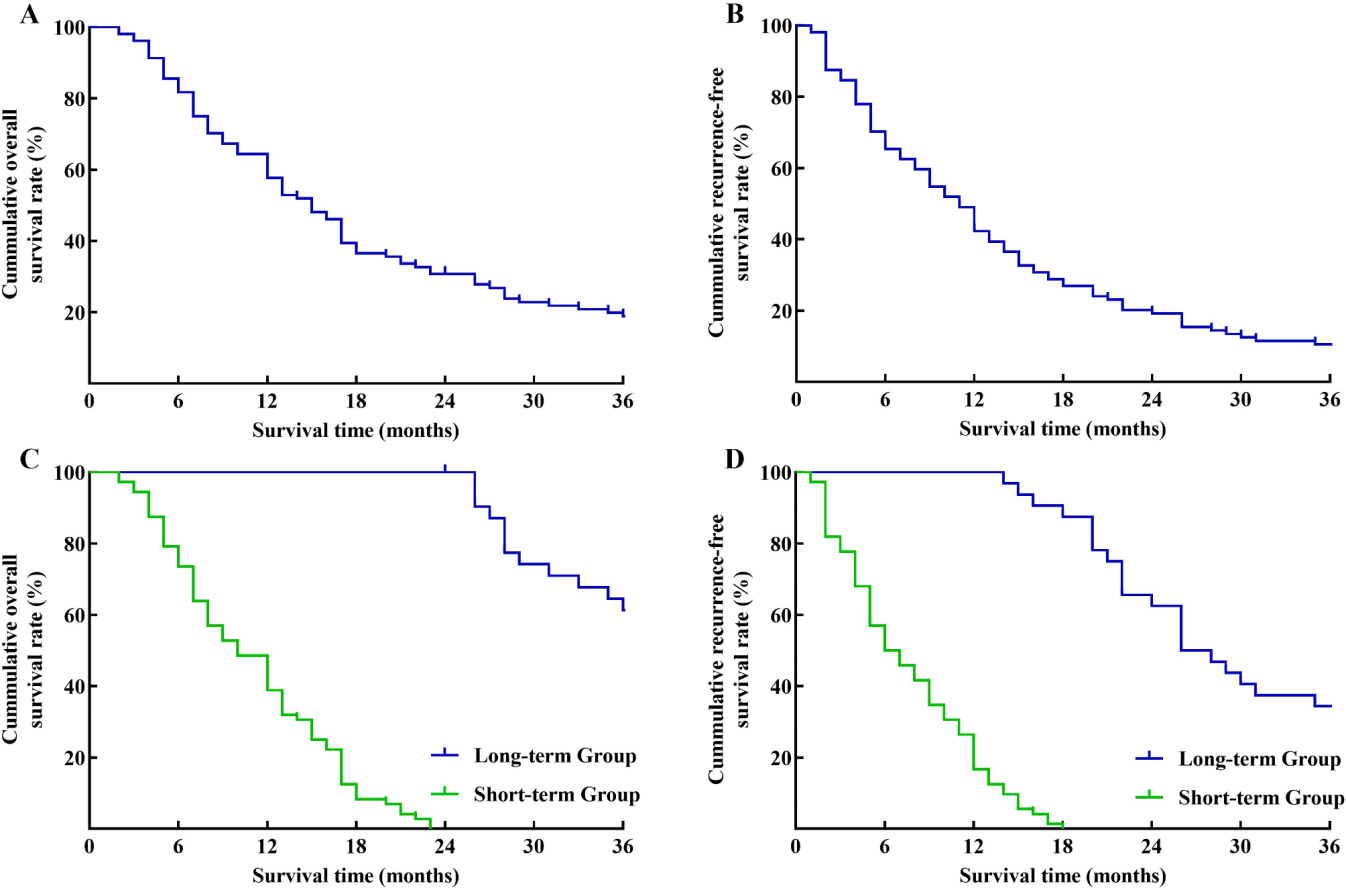
Survival curve of patients. (**A**) Overall survival (OS) curve of patients with BRPC. (**B**) Disease-free survival (DFS) curve of patients with BRPC. (**C**) OS curve between the long-term group and the short-term group. (**D**) DFS curve between the long-term group and the short-term group

A total of 104 patients were enrolled in the study, including 49 males and 55 females with an average age of 62.2 ± 10.2 years. The median OS was 15.5 months (Fig. 3A), and the rates of 0.5-, 1-, 2- year OS were 81.7%, 57.7%, and 30.8%. The median disease-free survival (DFS) was 11 months (Fig. 3B), and the rates of 0.5-, 1-, 2- year DFS were 65.4%, 42.3%, and 19.2%.

### Identification of risk factors related to long-term survival

Based on the OS, patients were divided into two groups: the long-term group (> 2 years, *n* = 32, 30.8%) and the short-term group (≤ 2 years, *n* = 72, 69.2%). In the long-term group and short-term group, the median OS and the rates of 1-, 2-, and 3- years OS were 38 months, 100%, 100%, 61.3%, and 10 months, 38.9%, 0%, and 0%, respectively. The DFS and the rates of 1-, 2-, 3- years DFS were 26 months, 100%, 62.5%, 34.4%, and 6 months, 16.7%, 0%, and 0%, respectively (Fig. 3C-D). Then univariate and multivariate analyses were applied to identify the risk factors related to long-term survival between the groups. In the univariate survival analysis, 7 variables, including age (*P* = 0.001), gender (*P* = 0.03), preoperative ALT (*P* = 0.01), tumor size (*P* = 0.02), vascular invasion length (*P* < 0.001), vascular morphological malformation (*P* = 0.01), and local lymphadenopathy (*P* = 0.004) were statistically different in the two groups (Table 1). In the multivariate survival analysis, 4 variables, including age (OR = 1.121, 95%CI: 1.049–1.199, *P* = 0.001), vascular invasion length (OR = 3.442, 95%CI: 1.700-6.967, *P* = 0.001), vascular morphological malformation (OR = 5.173, 95%CI: 1.448–18.484, *P* = 0.011) and local lymphadenopathy (OR = 4.444, 95%CI: 1.112–0.935, *P* = 0.035) were confirmed as independent risk factors for long-term survival of patients (Table 2).

**Table 1:** Univariate Analysis of Risk factors for BRCP patients between two groups

Variables	long-term group (*n* = 32)	short-term group (*n* = 72)	*P*-value
Gender			
(Male/Female)	10/22	39/33	0.03
Age (Year)	57.3 ± 10.8	64.4 ± 9.2	0.001
BMI (kg/m^2^)	23.3 (22.2, 26.9)	22.9 (20.6, 24.5)	0.13
Preoperative leucocytes			
(×10^9^ /L)	5.7 (4.6, 7.5)	5.4 (4.5, 6.6)	0.66
Preoperative neutrophil percent			
(%)	64.1 ± 9.7	62.9 ± 8.4	0.49
Preoperative albumin			
(g/L)	38.9 ± 6.6	37.3 ± 4.4	0.22
Preoperative AST			
(U/L)	39.0 (19.8, 131.8)	33.0 (18.3, 75.5)	0.30
Preoperative ALT			
(U/L)	76.0 (29.3, 182.3)	28.5 (17.0, 75.5)	0.01
Preoperative total bilirubin (umol/L)	32.2 (11.4, 137.3)	14.8 (9.7, 118.8)	0.17
Preoperative direct bilirubin (umol/L)	24.4(5.0, 124.3)	6.8(3.2 98.9)	0.12
Preoperative GGT			
(U/L)	278.5 (25.3, 493.5)	129.0 (19.0, 47.3)	0.25
Preoperative CEA			
(ng/ml)	2.1 (1.8, 4.4)	2.8 (1.6, 5.8)	0.29
Preoperative CA19-9			
(U/ml)	158.5 (45.1, 525.2)	288.7 (38.2, 1109.5)	0.35
Preoperative drainage for jaundice			
(Yes/No)	4/28	15/57	0.31
Tumor size			
(cm)	3.0 (2.5, 4.0)	4.0 (3.0, 5.0)	0.02
Tumor location of pancreas			
(Head and Neck/Body and Tail)	31/1	66/6	0.32
Portal vein confluence invasion			
(Yes/No)	11/21	31/41	0.59
Vascular invasion length			
(cm)	2.1 (1.7, 2.2)	3.2 (2.2, 4.3)	< 0.001
Vascular invasion angle (°)	129.2 (98.4, 239.1)	135.7 (111.2, 261.8)	0.24
Vascular morphological malformation (Yes/No)	9/23	40/32	0.01
Vascular lumen occlusion			
(Yes/No)	7/25	16/56	0.96
local lymphadenopathy			
(Yes/No)	8/24	40/32	0.004

**Table 2:** Multivariate analysis of independent risk factors for BRCP patients between two groups

Variables	RR value	95% CI	*P*-value	
Age		1.121	1.049–1.199	0.001
Gender		1.961	0.550-7.000	0.29
Preoperative ALT		0.966	0.991–1.001	0.12
Tumor size		1.241	0.759–2.029	0.38
Vascular invasion length		3.442	1.700-6.967	0.001
Vascular morphological malformation		5.173	1.448–18.484	0.011
local lymphadenopathy		4.444	1.112–0.935	0.035

### Establishment and evaluation of logistic regression

**Fig. 4:**
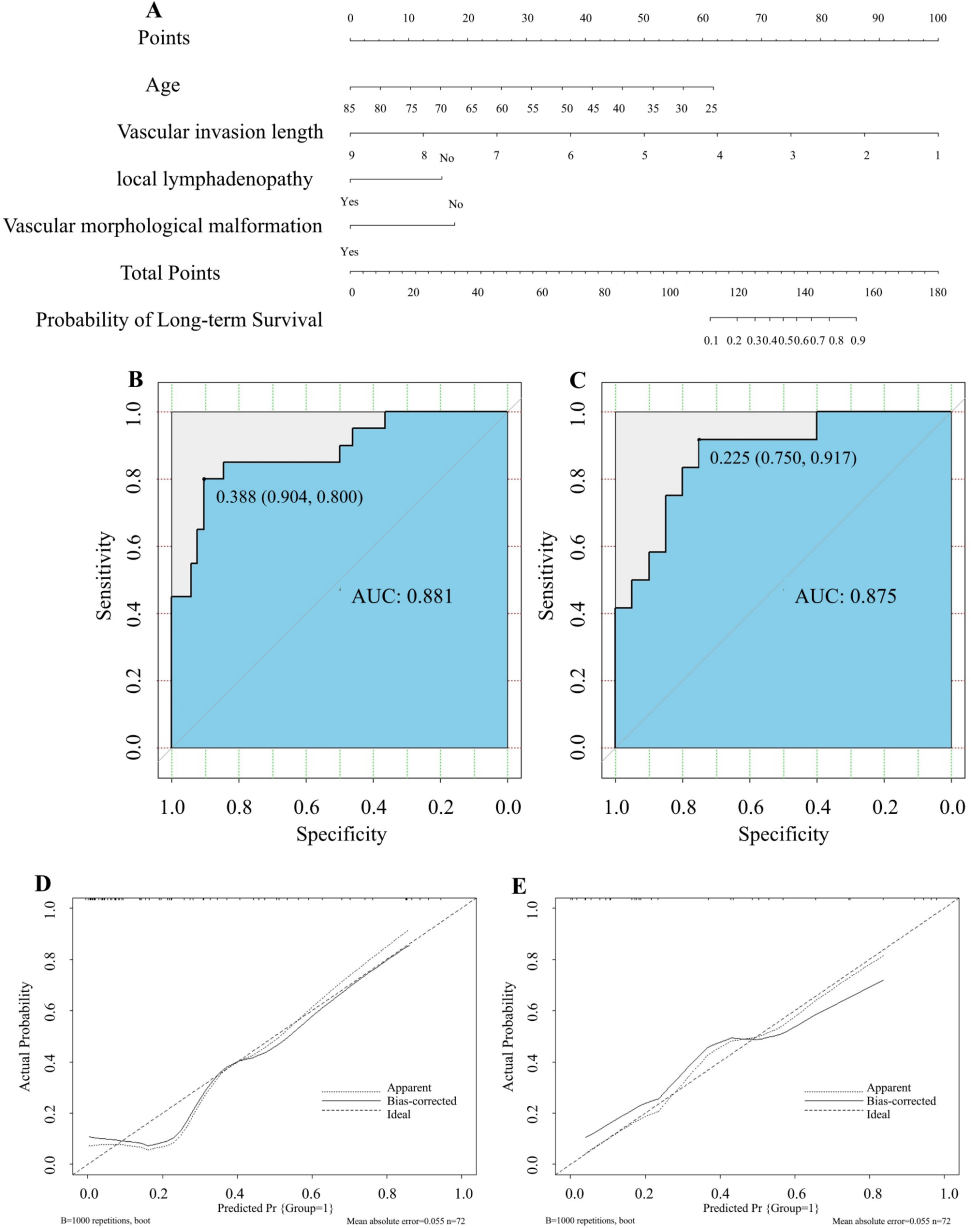
Establishment and evaluation of logistic regression. (**A**) A nomogram for predicting long-term survival in BRPC patients undergoing upfront surgery. (**B**) The ROC curve and the AUC value evaluate the discrimination ability of the nomogram in the training set. (**C**) The ROC curve and the AUC value evaluate the discrimination ability of the nomogram in the validation set. (**D**) Calibration curves for the training set. (**E**) Calibration curves for the validation set

All patients in the present study were randomly divided into the training set (*n* = 72) and the validation set (*n* = 32) at a 7:3 ratio. Based on the above four variables screened by logistic multivariate analysis, the logistic regression model was established and evaluated in the training set and the validation set, respectively, and was visualized by Nomograms (Fig. 4A), and its discrimination was evaluated by the AUC value of the ROC curve. The ROC curve in the training set with AUC: 0.881 (95%CI: 0.787–0.975), and the validation set with AUC: 0.875 (95%CI: 0.750-1.000), indicated that the model had a high discrimination (Fig. 4B-C). The calibration plots, which were used to assess the nomogram performance, demonstrated an excellent correlation between observed and predicted survival in both the training and validation sets with mean absolute errors of 0.054 and 0.054, respectively (Fig. 4D-E). The apparent curves of the model fit well with the bias-corrected curves, indicating a great agreement in the training and validation set.

### SHAP-based analysis of feature importance

**Fig. 5:**
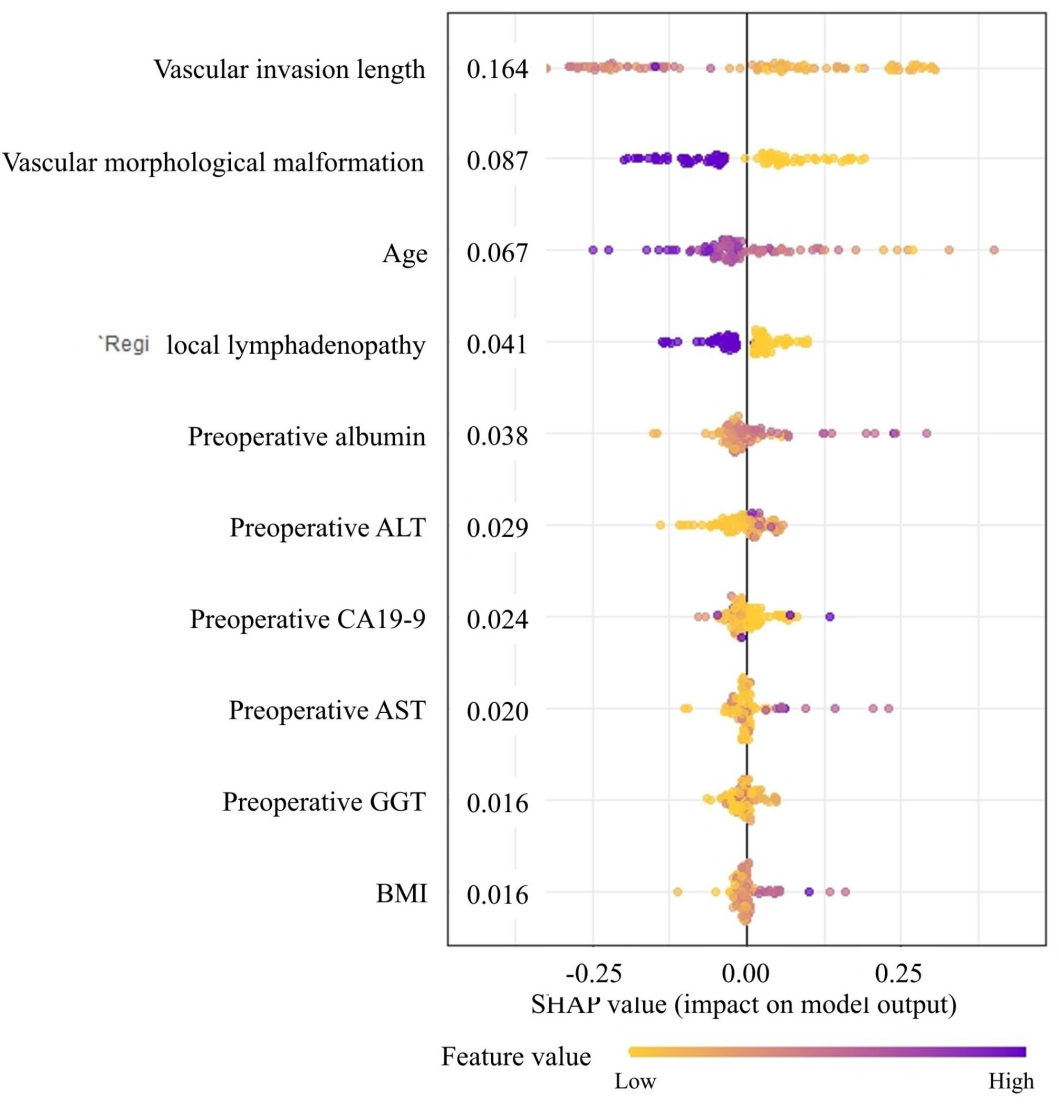
The SHAP summary plot for the top 10 important features in predicting long-term survival in patients

**Fig. 6:**
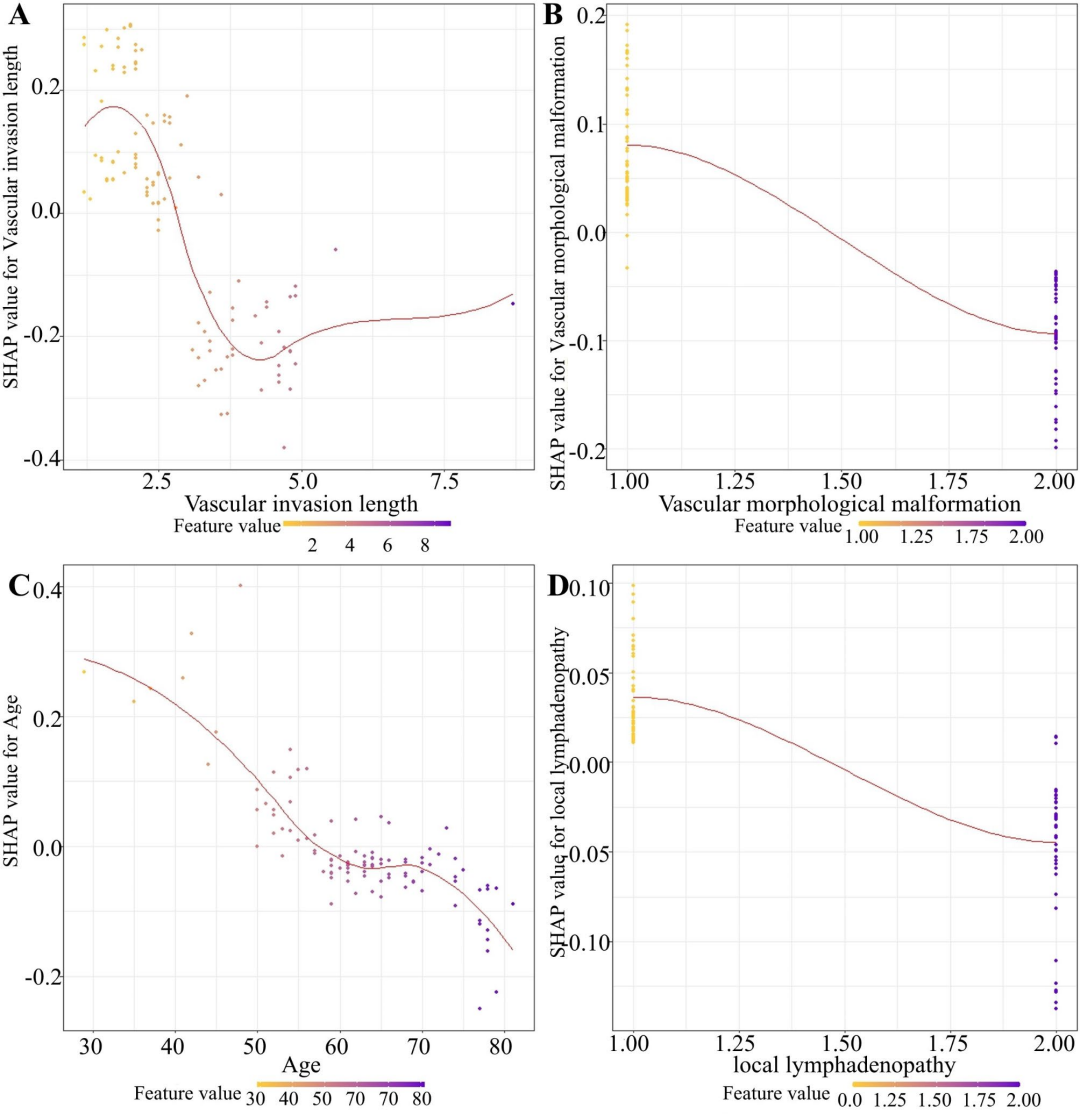
The SHAP dependence plots for the top 4 important features in predicting long-term survival in patients. (**A**) The SHAP dependence plots for the vascular invasion length in predicting long-term survival in patients. (**B**) The SHAP dependence plots for the vascular morphological malformation in predicting long-term survival in patients. (**C**) The SHAP dependence plots for the age in predicting long-term survival in patients. (**D**) The SHAP dependence plots for the local lymphadenopathy in predicting long-term survival in patients

Next, the SHAP value was performed to further identify the important variables that affect long-term survival postoperatively. The feature importance analysis revealed that vascular invasion length, vascular morphological malformation, age, and local lymphadenopathy were important factors affecting the long-term survival of BRPC patients following upfront surgery. As shown in Fig. 5, the figure illustrates the distribution of SHAP values for each feature, arranged in descending order according to the importance of each feature. The horizontal axis represents the SHAP value of the model, while the color of the dots indicates the magnitude of the feature value. Each point on the plot corresponds to a sample, and the horizontal axis indicates the SHAP value of a given feature across subjects, which reflects the magnitude of the SHAP value from low (yellow) to high (purple). As shown in Fig. 6, with the SHAP value as the vertical axis and the feature value as the horizontal axis, SHAP dependence plots make the values of many individuals available in one plot, facilitating an up-and-down trend of feature-attributed importance. Figure 6 shows the SHAP dependence plots for the top 4 features. The value on the horizontal axis represents the original value of a feature, whereas the value on the vertical axis represents the SHAP value of a feature across individuals. Those whose age scores of 50 exhibit higher SHAP values than those with scores of 70, indicating a higher likelihood of long-term survival prediction.

### Comparison of logistic regression with other machine learning algorithms

To evaluate the effectiveness of various machine learning algorithms in predicting the long-term survival of BRPC patients with upfront surgery, we also constructed other different predictive models, including support vector machine, random forest, decision tree, and XGBoost. 10-fold cross-validation as a statistical method and AUC value were used to estimate the general performance of machine learning models. The calculated results showed that the support vector machine model had the worst classification effect, with an average AUC value of 0.693, and the logistic regression model had the best classification effect, with an average AUC value of 0.864 (Table 3).

**Table 3:** Comparison of the effectiveness of various machine learning algorithms

Model	AUC
Logistic regression Random forest	0.864 0.789
Decision tree	0.790
Support vector machine	0.693
XGBoost	0.726

## Discussion

In recent years, there has been a growing interest in radical surgery combined with venous resection and reconstruction as a treatment for pancreatic cancer, due to advancements in comprehensive oncology techniques [16, 17]. Surgery remains the most effective way for pancreatic cancer patients to achieve long-term survival, with the portal vein system being the most commonly invaded vessel. Varadhachary et al. [18, 19] from the Anderson Cancer Center proposed criteria for BRPC in 2006, defining patients with vascular invasion of pancreatic cancer as suitable for surgical treatment. The American Hepatobiliary and Pancreatic Association issued an expert consensus related to BRPC in 2009 [20], which promoted radical pancreatic cancer surgery combined with portal vein system resection and reconstruction.

In the early 1950s, Moore et al. [21] performed resection and reconstruction of the invaded superior mesenteric vein in patients with BRPC, but with a poor prognosis. Fortner et al. [22] described a regional pancreatectomy in 1973 that included total pancreatectomy, radical lymph node dissection, combining portal vein resection and/or arterial resection and reconstruction, which was initially abandoned due to its lack of survival benefit, and higher complication rate and mortality than conventional surgery. However, with advances in surgical techniques, anesthesia, and intensive care support over the past decade, radical pancreatic cancer surgery with portal vein system resection and reconstruction has been becoming more widely used. Nonetheless, the conclusions of studies on invaded vein resection and reconstruction in international pancreatic centers are inconsistent. In 2008, Yekebas et al. [23] found that patients undergoing PD with invaded vein resection and reconstruction differ slightly in postoperative morbidity and mortality compared to those who didn’t undergo this operation. However, it has been shown that patients undergoing PD with invaded vein resection and reconstruction tend to have higher complication rates and no significant survival benefit. A systematic review study by Siriwardena et al. [24] showed that patients undergoing PD with invaded vein revascularization were significantly associated with lymph node metastasis and poor survival. Therefore, effective preoperative prediction of patient survival after upfront surgery can help improve clinical therapy options.

Numerous clinical studies have demonstrated that the prognosis of patients with BRPC is closely related to tumor characteristics, such as tumor size, degree of differentiation, depth of infiltration, nerve invasion, lymph node metastasis, and other factors. Imamura et al. [25] studied the perioperative predictors of BRPC in 225 patients treated with surgery and found that CA19-9 ≥ 37 U/ml and tumors larger than 3 cm were independent risk factors for early postoperative recurrence. Pancreatic cancer is also highly susceptible to peripheral nerve invasion due to its special anatomical location, where aggressive tumor cells invade the nerves and cause neurogenic inflammation. Neurons and surrounding glial cells can promote the development of pancreatic cancer through secretory signals, often predicting a poor prognostic outcome [26]. Furthermore, a study about prognostic factors for overall survival and recurrence-free survival of 351 patients with pancreatic cancer who underwent R0 resection and adjuvant therapy at Seoul National University Hospital showed that lymph node metastasis had a significant relationship with local area recurrence. Clinical features, such as surgical approach and postoperative adjuvant chemotherapy, likewise had a profound impact on patient prognosis [27].

The present study investigated the prognosis of patients with BRPC who underwent upfront surgery with portal-systemic resection and reconstruction, and its association with age. A stratified analysis of 280,000 pancreatic cancer patients in the United States from 2000 to 2018 revealed a higher prevalence of patients over the age of 55, who were often burdened with chronic disease and decreased physiological function, making post-surgical rehabilitation and treatment more challenging [28]. Consequently, the prognosis of older patients was inferior compared to their younger counterparts. However, a retrospective study by Dongen et al. found that younger patients had better OS, likely due to fewer underlying diseases and a higher willingness to receive surgery [29]. Nonetheless, survival rates were similar when stratified according to treatment, suggesting that the prognosis needs to be comprehensively evaluated in conjunction with other indicators.

In BRPC, preoperative vascular invasion is found in about 30–60% of patients, and about 10–50% have inseparable tumor adhesions in the portal vein system, necessitating resection of the invaded vessels for resection and reconstruction. This study systematically evaluated vascular invasion in BRPC and assessed its resectability by preoperative enhanced CT and/or MRI. Indicators such as the length of vascular invasion and the width of adipose tissue around the vessel were measured to determine the length and angle of vascular invasion, as well as preoperative imaging combined with pathological findings to determine whether the tumor had invaded the intima. Tran et al. conducted a detailed assessment of the angle of vascular invasion by abdominal CT examination before surgery, which was verified to have a high positive prediction rate (91.8%) and negative prediction rate (87.1%) by intraoperative exploration and postoperative pathological results [30]. The results of this study showed that an angle of vascular invasion ≤ 180° had better OS. Additionally, the length of vascular invasion and vascular endothelial invasion were significantly associated with patient prognosis. Ravikumar et al. analyzed the prognosis of 229 patients with pancreatic cancer who underwent portal vein systemic resection and reconstruction and found no significant difference in median OS between patients with superficial and deep veins and those without histological venous involvement [31]. Yu et al. analyzed 22 retrospective studies involving 2890 patients in different histopathological subgroups and found that patients with venous tumor infiltration had significantly lower 1- and 5-year survival rates compared to those with inflammatory pathology only, similar to the findings of this study [32]. This may be because intimal invasion indicates that the tumor has spread to the hematologic system and makes it more susceptible to metastasis and recurrence.

According to data from the 2012–2014 US and European pancreatic cancer registries, a mere 13–21% of pancreatic cancer patients had access to surgical resection. Even for patients with stage I-II pancreatic cancer, the 3-year survival rate was merely 17–39% [33]. A global study of 168,949 patients who received chemotherapy and pancreatic cancer surgery revealed that the median survival time postoperatively was 23 months for US patients and 18 months for Slovenian patients [34]. Against this backdrop, the present study aimed to develop a prediction model for the long-term survival of patients with BRPC after direct surgery with invasive vein resection and reconstruction. Notably, the preoperative single index is unreliable for predicting the postoperative prognosis of patients with BRPC. Thus, we established a logistic regression model incorporating four factors: age, vascular invasion length, tunica intima invasion, and postoperative chemotherapy. The logistic regression model obtained high AUC in both the training and validation sets, indicating its high discrimination. Furthermore, SHAP was introduced for visualization and analysis of the impact of major clinical factors on the model, listing the top 6 important features of all the variables. The predictive performance of the logistic regression prediction model for long-term survival postoperatively was comparable to that of traditional machine learning algorithms, demonstrating high accuracy and differentiation. Therefore, the present predictive model can be effectively applied in clinical work to assist clinicians in making appropriate treatment decisions.

However, this study has certain limitations, including its relatively small sample size and single-center design. Although the logistic regression model was internally validated, the study results may still be biased. Thus, we suggest that future studies should enroll larger sample sizes and conduct multicenter studies using external data for further validation.

## Conclusions

In summary, this study has identified independent risk factors that affect long-term survival for patients with BRPC after upfront surgery with invaded vein resection and reconstruction. We also developed a reliable logistic regression model that can assist clinicians in making treatment decisions.

## Data Availability

No datasets were generated or analysed during the current study.

## Abbreviations

PCa: Pancreatic carcinoma
BRPC: Borderline resectable pancreatic cancer
PD: Pancreaticoduodenectomy
CT: Computed tomography
PDAC: Pancreatic ductal adenocarcinoma
ROC: Operating characteristic curve
C-index: Consistency index
DCA: Decision curve analysis
SHAP: Shapley additive explanation
DFS: Disease-free survival
OS: Overall survival
AUC: Area under curve
